# Transcriptome profiling of patient-derived tumor xenografts suggests novel extracellular matrix-related signatures for gastric cancer prognosis prediction

**DOI:** 10.1186/s12967-023-04473-0

**Published:** 2023-09-19

**Authors:** Ziqian Deng, Ting Guo, Jiwang Bi, Gangjian Wang, Ying Hu, Hong Du, Yuan Zhou, Shuqin Jia, Xiaofang Xing, Jiafu Ji

**Affiliations:** 1 Key Laboratory of Carcinogenesis and Translational Research (Ministry of Education/Beijing), Division of Gastrointestinal Cancer Translational Research Laboratory, Peking University Cancer Hospital and Institute, Beijing, 100142 People’s Republic of China; 2 Biological Sample Bank, Peking University Cancer Hospital and Institute, Beijing, 100142 People’s Republic of China; 3 Department of Biomedical Informatics, School of Basic Medical Sciences, Peking University, Beijing, 100191 People’s Republic of China; 4 Department of Molecular Diagnosis, Peking University Cancer Hospital and Institute, Beijing, 100142 People’s Republic of China; 5 Department of Gastrointestinal Surgery, Peking University Cancer Hospital and Institute, Beijing, 100142 People’s Republic of China

**Keywords:** Gastric cancer, Patient-derived tumor xenografts, Extracellular matrix, Prognosis

## Abstract

**Background:**

A major obstacle to the development of personalized therapies for gastric cancer (GC) is the prevalent heterogeneity at the intra-tumor, intra-patient, and inter-patient levels. Although the pathological stage and histological subtype diagnosis can approximately predict prognosis, GC heterogeneity is rarely considered. The extracellular matrix (ECM), a major component of the tumor microenvironment (TME), extensively interacts with tumor and immune cells, providing a possible proxy to investigate GC heterogeneity. However, ECM consists of numerous protein components, and there are no suitable models to screen ECM-related genes contributing to tumor growth and prognosis. We constructed patient-derived tumor xenograft (PDTX) models to obtain robust ECM-related transcriptomic signatures to improve GC prognosis prediction and therapy design.

**Methods:**

One hundred twenty two primary GC tumor tissues were collected to construct PDTX models. The tumorigenesis rate and its relationship with GC prognosis were investigated. Transcriptome profiling was performed for PDTX-originating tumors, and least absolute shrinkage and selection operator (LASSO) Cox regression analysis was applied to extract prognostic ECM signatures and establish PDTX tumorigenicity-related gene (PTG) scores. The predictive ability of the PTG score was validated using two independent cohorts. Finally, we combined PTG score, age, and pathological stage information to establish a robust nomogram for GC prognosis prediction.

**Results:**

We found that PDTX tumorigenicity indicated a poor prognosis in patients with GC, even at the same pathological stage. Transcriptome profiling of PDTX-originating GC tissues and corresponding normal controls identified 383 differentially expressed genes, with enrichment of ECM-related genes. A robust prognosis prediction model using the PTG score showed robust performance in two validation cohorts. A high PTG score was associated with elevated M2 polarized macrophage and cancer-associated fibroblast infiltration. Finally, combining the PTG score with age and TNM stage resulted in a more effective prognostic model than age or TNM stage alone.

**Conclusions:**

We found that ECM-related signatures may contribute to PDTX tumorigenesis and indicate a poor prognosis in GC. A feasible survival prediction model was built based on the PTG score, which was associated with immune cell infiltration. Together with patient ages and pathological TNM stages, PTG score could be a new approach for GC prognosis prediction.

**Supplementary Information:**

The online version contains supplementary material available at 10.1186/s12967-023-04473-0.

## Background

Gastric cancer (GC) is the third leading cause of cancer-related deaths in China. [1, 2] Approximately 44% of GC cases and 48.6% of GC mortalities have been reported in China [1, 3]. Additionally, over the past 35 years in China, the incidence and mortality of GC have remained high, with an overall 5-year survival rate of less than 50% [4]. One of the crucial barriers to GC-targeted therapies is intra-tumor, intra-patient, and inter-patient heterogeneity [5, 6]. Fortunately, patient-derived tumor xenograft (PDTX) models can recapitulate the molecular diversity of donor tumors, making in vivo translational research feasible [7–9]. Therefore, PDTX models are now widely used in biomarker development, preclinical drug screening, and personalized clinical decision making [7–9]. In addition, PDTX models can be used to investigate the biological functions of donor tumors.

The tumor microenvironment (TME) is comprised of extracellular matrix (ECM), blood vessels, neurons, immune cells, cancer-associated fibroblasts (CAFs), and other non-malignant cells, and it plays a key role in regulating tumor cell proliferation, drug resistance, and metastasis [10–12]. Among the various components of the TME, the ECM plays a major role. The ECM is a complex system that provides mechanical support for the TME, modulates growth factor secretion, and mutually interacts with tumor and immune cells in the TME [13–16]. Specifically, the ECM contains various macromolecules with distinctive physical, biochemical, and biomechanical properties. During tumorigenesis, the ECM system is deregulated to favor the generation of a tumorigenic microenvironment that enhances tumor-associated angiogenesis and inflammation [15]. The ECM goes through remodeling during tumor development. This remodeling contributes to the establishment of a premetastatic niche by reorganizing or degrading the pre-existing matrix architecture or by stimulating local matrix secretion [17]. Accordingly, the investigation of ECM-related molecular characteristics is of great value for personalized GC treatment.

In general, GC stages and histotypes are diagnosed based on morphological characteristics at the cellular or tissue level [2]. In clinical practice, only a few molecular markers are commonly examined using immunohistochemistry (IHC) and fluorescence in situ hybridization (FISH) for treatment guidance. According to the National Comprehensive Cancer Network (NCCN) and The Chinese Society of Clinical Oncology (CSCO) clinical guidelines [18, 19], human epidermal growth factor receptor 2 (HER2) status testing is recommended for all patients with GC; programmed death protein-1 (PDL1) expression and microsatellite instability (MSI)/mismatch repair (MMR) status evaluation is recommended for patients with GC who are to undergo immunotherapy; and neurotrophic tropomysin-related kinase (NTRK) gene fusion testing is recommended for patients with GC who have failed to respond to standard treatment. In addition to these routinely tested molecules, several genomic classifications have been reported, including Epstein-Barr virus (EBV)-positive GC, characterized as EBV-encoded-RNA-positive [20]. However, such a small number of molecular tests is not sufficient to help clinicians fully understand the transcriptomic characteristics of patients with GC. In addition, IHC and FISH can only achieve a semi-quantitative determination of molecular expression levels, and they do not provide precise expression information for these markers. However, GC is well known for its intra-tumor and inter-individual heterogeneity [5], and prognosis predictions that simply rely on morphological and histological information may result in inaccurate prognosis prediction and failure in post-surgical treatment.

Recent research has shown that, even in patients at the same stage and subtype, the transcriptomic landscapes of primary tumors can be distinguished from one another [21]. We noted a divergence in tumorigenic potential among GC patients with the same clinical characteristics and pathological stage diagnosis. Because PDTX models recapitulate the molecular diversity of donor tumors, they mimic the inter-individual heterogeneity seen in patients with GC. Therefore, we investigated whether heterogeneity exists at the transcriptomic level among these patients and whether gene expression signatures can be adopted to improve GC prognosis. Our findings will be helpful in the prediction of GC prognosis and will provide new insights into research on therapeutic targets for GC.

## Methods

### PDTX model construction

Fresh GC and adjacent normal control tissues (at least 2 cm from the matched GC tissues) were surgically resected. According to previously reported methods [22], GC tissues were cut into 3 mm^3^ pieces and implanted subcutaneously in 6-week-old non-obese diabetes/severe combined immunodeficiency (Nod/Scid) mice. We observed and recorded the tumor size in the mice every 3 days. When the tumor size increased to 1000 mm^3^, we stopped our observation and harvested the tumor for further study. Implanted tumors that showed no growth within 6 months were categorized into the non-tumorigenesis group.

### Sample and data collection

In total, 1003 GC samples with RNA expression data and clinicopathological information from four cohorts (n = 122, 300, 387, and 194) were included in this study. Of these, 122 samples used for PDTX model construction were treated at PKUCH (Beijing, China). The Asian Cancer Research Group (ACRG) cohort, which consists of 300 cancer samples, was used as the training cohort [23], and all 300 samples were used for model construction. The Cancer Genome Atlas (TCGA) stomach adenocarcinoma (STAD) cohort and 198 patients with GC treated at PKUCH were used as validation cohorts [24]. All 387 samples in the TCGA cohort and 194 samples in the PKUCH validation sets, with available overall survival (OS) data, were used for OS analysis and 304 samples in the TCGA cohort and 194 samples in PKUCH, with available progression-free survival (PFS) data, were used for PFS analysis. RNA expression data and clinical information for patients in the TCGA cohort were downloaded from the University of California Santa Cruz (UCSC) website (https://xenabrowser.net/datapages/, accessed on February 28, 2023). Correlated data for the ACRG cohort were downloaded from the Gene Expression Omnibus (GEO) database (https://www.ncbi.nlm.nih.gov/geo/, accessed on February 28, 2023). The 5-year survival and clinical data for the TCGA cohort were collected from the UCSC website (https://xenabrowser.net/datapages/, accessed on February 28, 2023). The 5-year survival and clinical data for the ACRG cohort were collected from supplementary data files in the literature [23]. The 5-year survival and clinical data for the PKUCH cohort were provided by the corresponding author [24]. All patients provided written informed consent and the Institutional Review Board of PKUCH approved this study (2019KT11).

### Whole tissue RNA-sequencing

1. RNA extraction
  Total RNA was extracted from the tissues using TRIzol Reagent (Invitrogen Life Technologies, Carlsbad, CA, USA) according to the manufacturer’s instructions. The RNA quality and integrity were determined using a NanoDrop spectrophotometer (Thermo Fisher Scientific, Waltham, MA, USA). Only high-quality RNA samples (OD_260/280_ = 1.8~2.2, OD_260/230_ ≥ 2.0) were used to construct the sequencing library.
2. Library preparation and sequencing
  The library was prepared using 3 μg of total RNA. First, mRNA was isolated according to the polyA selection method using oligo(dT) beads, and then fragmented using divalent cations at elevated temperatures in a proprietary fragmentation buffer (Illumina, San Deigo, CA, USA). Second, first-strand cDNA was synthesized using random oligonucleotides and Super Script II, and second-strand cDNA synthesis was subsequently performed using DNA polymerase I and RNase H. The remaining overhangs were then converted into blunt ends via exonuclease/polymerase activities, after which, the enzymes were removed. After adenylation of the 3′ ends of the DNA fragments, Illumina paired-end adapter oligonucleotides were ligated to prepare them for hybridization. Next, to select cDNA fragments of the preferred size (400–500 bp), the library fragments were purified using an AMPure XP system (Beckman Coulter, Brea, CA, USA). DNA fragments with ligated adaptor molecules at both ends were selectively enriched using an Illumina PCR Primer Cocktail in a 15 cycle PCR reaction. The products were purified (AMPure XP system) and quantified using an Agilent High-Sensitivity DNA Assay on a Bioanalyzer 2100 system (Agilent Technologies, Santa Clara, CA, USA). Finally, paired-end RNA-sequencing (RNA-seq) libraries were sequenced using a NovaSeq 6000 sequencer (Illumina; 2 × 150 bp read length).
3. Quality control and read mapping
  Quality control was performed on raw paired-end reads using FastQC (v0.11.9, https://www.bioinformatics.babraham.ac.uk/projects/fastqc/), with default parameters, and trimmed using Trim Galore (v0.6.7, https://www.bioinformatics.babraham.ac.uk/projects/trim_galore/), also with default parameters. The clean reads were aligned to the reference genome (hg38) using STAR software [25]. Next, genes were annotated using the “gencode.v38.primary_assembly.annotation.gtf” file downloaded from the GENCODE website (https://www.gencodegenes.org) and gene expression levels were quantified using RSEM software, with the default parameters [26]. Raw RNA-seq datasets (fastq files) were uploaded to the National Genomics Data Center (NGDC, https://ngdc.cncb.ac.cn) under accession number HRA004403.
4. Differential expression analysis and functional enrichment
  To identify differentially expressed genes (DEGs) between GC and normal samples, differential expression analysis was performed using the DESeq2 package. DEGs with |log_2_ fold change| ≥ 0.678 and *P* < 0.05 were considered to be significant. In addition, functional enrichment analysis of Gene Ontology (GO) terms was performed with a Benjamini-and-Hochberg-corrected *P*-value threshold of 0.01 and an overlapped gene number threshold of 10. GO functional enrichment analysis was performed using KOBAS [27].

### STRING protein–protein interaction analysis

DEGs were submitted to the STRING database [28] to identify associated genes (90% confidence, 5% false discovery rate). Interactions were examined using curated databases, experimentally determined gene neighborhoods, gene fusions, gene co-occurrence, text mining, co-expression, and protein homology.

### Establishment and validation of the PDTX-tumorigenicity-related gene score for GC prognosis prediction

In the training set, 383 unique DEGs between GC and normal tissues in the tumorigenesis samples were included to train a univariate Cox regression model using the “survival” R package [29]. In total, 116 candidate prognostic genes were identified. Next, least absolute shrinkage and selection operator (LASSO) Cox regression models were used to identify the most robust markers related to survival. Four PDTX tumorigenicity-related genes (PTGs) were integrated to construct a predictive signature for the PTG score.

$$PTG\,score=\sum \left(LASSO\,coefficient\,of\,RNAi\times\,RNAi\,expression\right).$$

The best PTG score cut-off value was determined by the median value to enhance accuracy. The samples were classified into low- and high-PTG groups based on their median values. We then compared OS and PFS between the two groups to validate the prognostic predictive values using the ACRG, TCGA and PKUCH validation cohorts.

### Characterization of immune cell infiltration

To depict the immune cell infiltration landscape of GC patients with low and high PTG scores, the CIBERSORTx algorithm was used to assess the abundance of 22 immune-infiltrating cells [30]. The TIMER2.0 database (http://timer.cistrome.org) was used to estimate the proportion of immune cell infiltration.

### Stromal and immune score analysis

To evaluate the stromal and immune scores of GC samples, we employed the ESTIMATE (Estimation of STromal and Immune cells in MAlignant Tumors using Expression data) algorithm using the “estimate” R package [31]. The correlation between the PTG score and stromal score, the ESTIMATE score, and tumor purity were visualized using the “ggplot2” R package.

### Establishment and validation of the integrative nomogram

Multivariate Cox regression analysis was used to assess the correlation between clinicopathological features and PTG scores in the ACRG cohort. Based on the multivariate analysis results, the significant factors (*P* < 0.05) were subsequently used as inputs to develop a predictive nomogram utilizing the “rms” R package. The predictive accuracy of the nomogram scoring system was evaluated by receiver operating characteristic (ROC) analysis for 1-, 3-, and 5-year survival rates. Calibration curves were used to depict the consistency between predicted survival events and actual observations.

### Statistical analysis

Student’s t-tests were used to compare continuous variables between the two groups, and chi-square tests were used to compare categorical variables. The Kaplan–Meier method was used to compare the OS and PFS between patient subgroups. Univariate and multivariate Cox regression models were used to evaluate the independent prognostic value of the PTG signature. A *P* ≤ 0.05 was considered a statistical significance. All statistical analyses were performed using R software (v4.2.2).

## Results

### PDTX tumorigenicity indicates poor prognosis in patients with GC

**Fig. 1:**
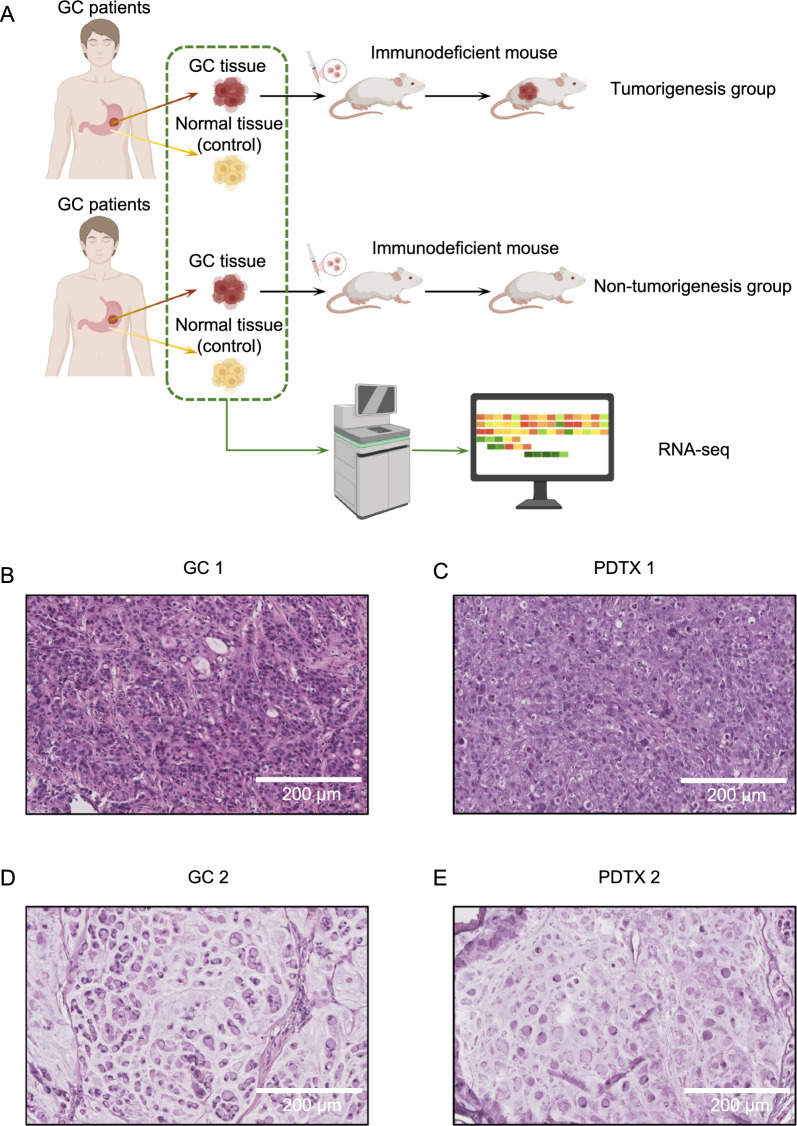
Outline of the study flow. **A** Schematic diagram of the study flow. **B**, **D** Representative hematoxylin–eosin (HE) staining of gastric cancer (GC) tissue from the tumorigenesis group. **C**, **E** Representative HE staining of patient-derived tumor xenograft (PDTX) tumor tissue

**Fig. 2:**
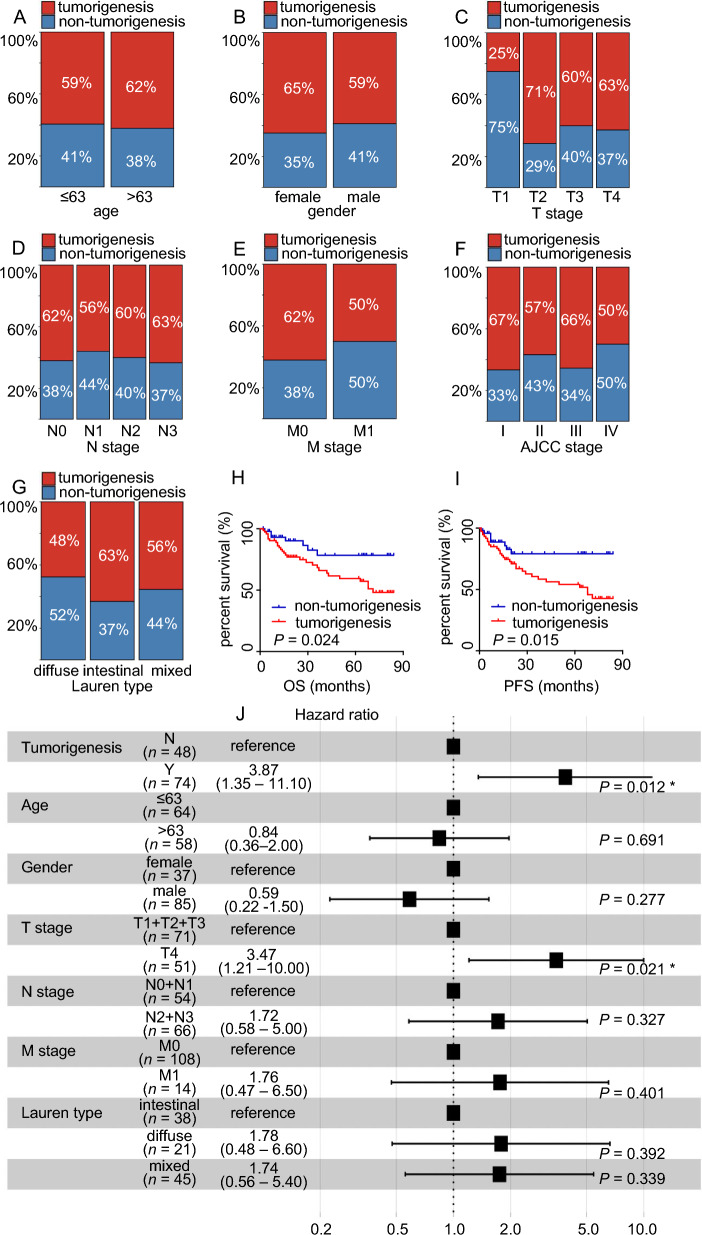
PDTX models of tumorigenicity correlate with GC patient survival. **A**–**G** Correlation analysis of PDTX tumorigenesis and clinical characteristics. **H**, **I** Kaplan–Meier curves for overall survival (OS) and progression-free survival (PFS) of 122 GC patients. **J** Multivariate Cox regression analysis of tumorigenicity and clinical characteristics of GC. *P < 0.05, **P < 0.01, ***P < 0.001, ****P < 0.0001

We collected 122 GC cases to construct the PDTX model in Nod/Scid mice. Of the 122 cases, 74 successfully developed tumors in mice, namely the tumorigenesis group, and 48 failed to develop tumors, namely the non-tumorigenesis group (Fig. 1A–E; Additional file 1: Fig. S1). We then investigated the patient composition of the two groups (Fig. 2A–G). However, there were no significant differences in the clinical characteristics between the groups. Interestingly, of the four patients at the T1 stage, only one was in the tumorigenesis group. This suggests that GC at the T1 stage may not be suitable for PDTX-model-based investigations. We explored the prognosis of all 122 patients. Patients whose tumor samples successfully developed the PDTX model exhibited a poor prognosis (Fig. 2H–I). Next, to rule out the effects of tumor stage and patient age on prognosis, we performed prognosis analysis for each TNM stage. The results showed that, even at the same stage or within the same age range, patients with successful tumorigenicity exhibited poorer prognosis (Additional file 1: Fig. S2). Univariate and multivariate Cox regression analyses were performed to explore the prognostic predictive ability of tumorigenicity. The results showed that tumorigenicity was an independent risk factor in patients (Fig. 2J; Additional file 2: Table S1). Therefore, we hypothesized that the PDTX model could be used as an in vivo tool for predicting GC prognosis (Table 1).

**Table 1:** Clinical characteristics of 122 patients with GC for PDTX models

Characteristics	N (%)
Gender	
Male	85 (69.7)
Female	37 (30.3)
Age	
Male median age [range]	63 [28–80]
Female median age [range]	63 [26–85]
Location	
Proximal stomach	17 (13.9)
Gastric body	69 (56.6)
Distant stomach	36 (29.5)
Differentiation	
Moderate/poor	120 (98.4)
Well	2 (1.6)
Tumor stage	
I	6 (4.9)
II	44 (36.1)
III	58 (47.5)
IV	14 (11.5)
T stage	
T1	4 (3.3)
T2	7 (5.7)
T3	60 (49.2)
T4	51 (41.8)
N stage	
N0	29 (23.8)
N1	25 (20.5)
N2	25 (20.5)
N3	41 (33.6)
Nx	2 (1.6)
M stage	
M0	108 (88.5)
M1	14 (11.5)
Lauren classification (n = 104)	
Intestinal	38 (36.5)
Diffuse	21 (20.2)
Mixed	45 (43.3)

However, owing to the limited amount of GC tissue available for diagnosis, it is not feasible to perform a PDTX model test for every GC patient. Therefore, we wondered if there were unique molecular signatures in PDTX-originating tumor (POT) tissues that regulate tumor cell tumorigenesis and can be applied for GC prognosis prediction.

### ECM-related gene signatures contribute to tumorigenicity

**Fig. 3:**
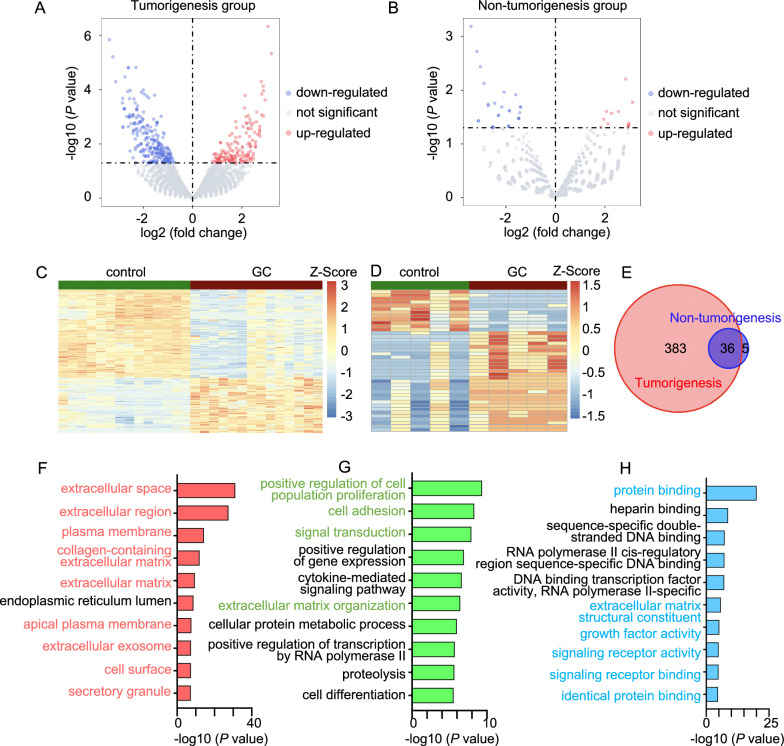
Transcriptome profiling of PDTX-model-originating patients. **A**, **B** Differentially expressed genes (DEGs) between GC tissue and normal tissue in the tumorigenesis group (**A**) and non-tumorigenesis group (**B**). Red dots represent highly expressed genes in GC tissue with *P* < 0.05 and a fold change > 1.6, and blue dots represent down-regulated genes in GC tissue with *P* < 0.05 and a fold change < 0.625. **C**, **D** Heatmap of DEG expression levels in the tumorigenesis group (**C**) and non-tumorigenesis group (**D**). **E** Venn diagram of DEGs in the tumorigenesis and non-tumorigenesis group. **F**–**H** Gene Ontology (GO) enrichment bar plots of 383 unique DEGs in the tumorigenesis group for cellular component (red), biological process (green), and molecular function (blue) terms. Colored titles are extracellular matrix (ECM)-related pathways

To uncover the underlying molecular signatures in POT tissues, we performed RNA-seq analysis of POT tissues (GC) and their corresponding normal tissues (control) from 19 patients (Fig. 1A; Additional file 2: Tables S2–S3). Compared to the normal control tissue, we identified 419 DEGs in the GC tissue of the tumorigenesis group and 41 DEGs in the GC tissue of the non-tumorigenesis group (Fig. 3A–D). By overlapping the two groups of DEGs, we found 383 unique DEGs in the tumorigenesis group, but only five unique DEGs in the non-tumorigenesis group. To explain the stark contrast between the numbers of unique DEGs in these two groups, we inferred that there was less molecular heterogeneity between GC and control tissues in the non-tumorigenesis group. Furthermore, we further investigated the five unique DEGs in the non-tumorigenesis group to identify possible connections among them. The five DEGs identified were *CENPF* (centromere protein F), *LIPF* (lipase F, gastric type), *PELATON* (plaque enriched long non-coding RNA (lncRNA) in atherosclerotic and inflammatory bowel macrophage regulation), *PRRT3-AS1* (*PRRT3* antisense RNA1), and *SMKR1* (small lysine rich protein 1). We performed protein–protein interaction analysis of the three protein-coding genes [28], and the results showed no significant interaction between them (Additional file 1: Fig. S3A). We found no published evidence for the association of the lncRNAs, *PELATON* and *PRRT3-AS1*, with GC survival; therefore, we did not focus on these five DEGs that were only found in the non-tumorigenesis group. In addition, there were 36 DEGs in both the tumorigenic and non-tumorigenic groups. To examine the molecular functions of these 36 DEGs, we performed GO enrichment analysis of biological processes, cellular components, and molecular function terms. Nineteen terms showed significant corrected enrichment *P*-values (Additional file 1: Fig. S3B). The enriched biological processes, cellular components, and molecular functions can be summarized into the following five main categories: ion transport and homeostasis, steroid-regulated pathways, digestion, creatine metabolism, and aspartic-type endopeptidase activity. These terms are highly related to cell metabolism, which implies that these 36 DEGs may play important roles in maintaining homeostasis in primary GC cells, but do not determine the tumorigenic ability of these cells in mice.

To further investigate the molecular signatures contributing to a poor prognosis of GC, we used the 383 unique DEGs in the tumorigenesis group (Fig. 3E) for GO enrichment analysis. As shown in the enrichment plots, of the top 10 enriched pathways in cellular component, biological process, and molecular function terms, 19 ECM-related pathways showed significant corrected enrichment *P*-values (Fig. 3F–H). ECM signatures in cancer have been reported to be responsible for regulating the immune response, metabolism, intracellular signaling, EMT, and metastasis [17], which strongly suggests that the ECM of GC may contribute to tumorigenesis in PDTX models. However, the GO terms of extracellular space, plasma membrane, and identical protein binding were also enriched for 36 DEGs (Additional file 1: Fig. S3B); therefore, rather than including all genes of the 19-ECM related pathways, we only selected 299 DEGs from the 383 unique DEGs enriched in the 19 ECM-related terms for further analysis.

### Construction of PTG-related prognosis signatures in GC

**Fig. 4:**
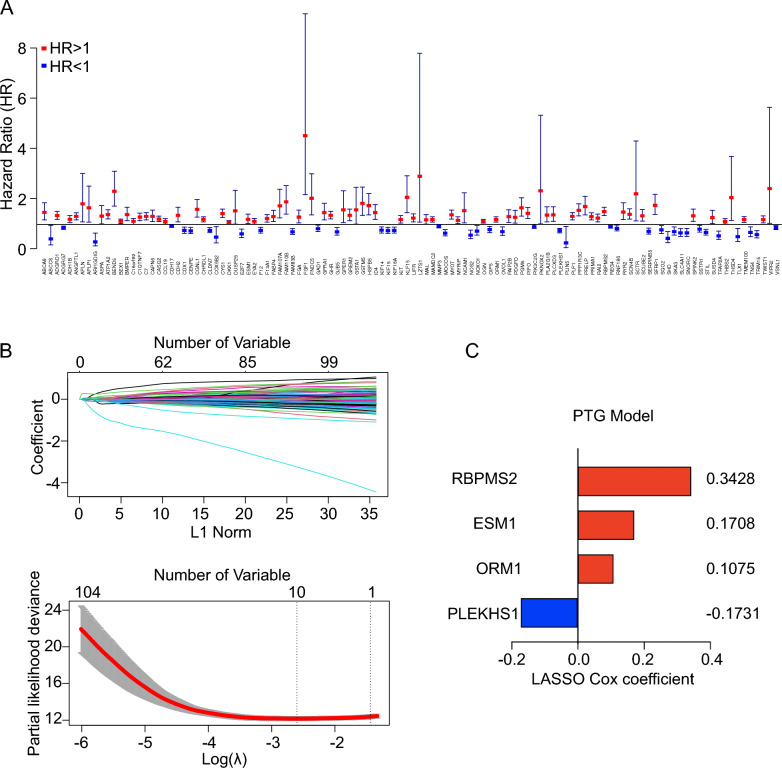
Construction of PTG signatures. **A** The hazard ratio of 116 candidate prognostic genes in the Asian Cancer Research Group (ACRG) cohort. **B** The most robust predictive genes were identified by the least absolute shrinkage and selection operator (LASSO) Cox regression algorithm. **C** An ensemble of four genes remained with non-zero LASSO Cox coefficients. PTG, PDTX-tumorigenicity-related gene

Due to the limited sample size of our RNA-seq data, we chose the ACRG cohort dataset to perform Cox regression analysis to extract ECM-related gene expression signatures. Using a univariate Cox regression analysis, we identified 116 candidate genes associated with GC prognosis (Fig. 4A). The expression levels of 116 candidate genes were highly correlated (Additional file 1: Fig. S4A), which implied the probability of variable interaction effects among these genes and intermolecular influences in predicting prognosis. LASSO Cox regression analysis was performed to identify the most robust and non-redundant genes that predicted GC prognosis (Fig. 4B). Finally, four genes (*RBPMS2*, *ORM1*, *ESM1*, and *PLEKHS2*) with individual non-zero LASSO regression coefficients were screened and integrated to establish a PTG score model (Fig. 4C).

**Fig. 5:**
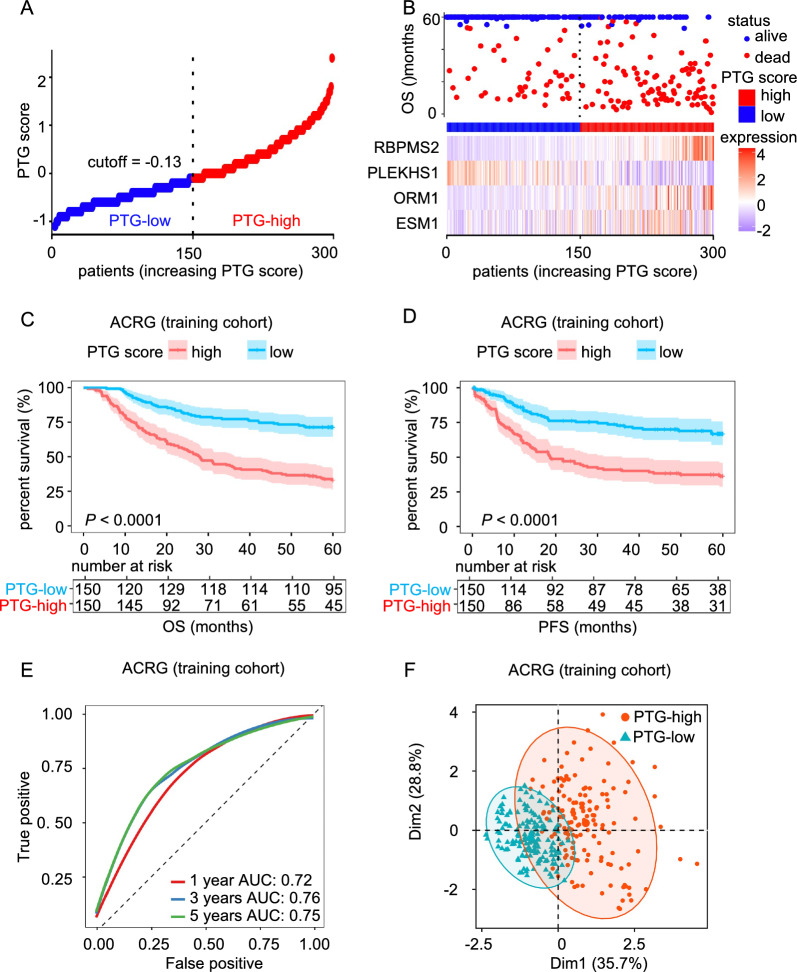
Prognosis analysis in the training cohort. **A**, **B** The distribution and median values of PTG scores. **C**, **D** Kaplan–Meier curves for the OS and PFS of GC patients in the low and high PTG score groups. **E** Area under the curve (AUC) of time-dependent receiver operating characteristic (ROC) curves at 1, 3 and 5 years. **F** Principal component analysis (PCA) analysis showed different distribution patterns in the low and high PTG score groups. The ellipse represents the 95% confidence interval

To measure the prognostic value of our PTG signature, we investigated the relationship between PTG scores and 5-year OS in an independent ACRG cohort. With the median PTG score set as the cutoff, patients were assigned to high-risk or low-risk group (Fig. 5A). In the validation cohort, high PTG scores were associated with poor survival (Fig. 5B). Kaplan–Meier curves showed that patients with higher PTG scores had poorer OS and PFS (Fig. 5C–D). The areas under the ROC curve (AUCs) of the PTG score for 1-, 3-, and 5-year OS were 0.72, 0.76, and 0.75, respectively (Fig. 5E). In addition, to highlight the differences in the expression patterns of PTG-related genes, we performed principal component analysis (PCA) based on the PTG-related genes of the low- and high-risk groups. The scatter plot showed that the PTG expression patterns were substantially different between the two groups (Fig. 5F).

**Fig. 6:**
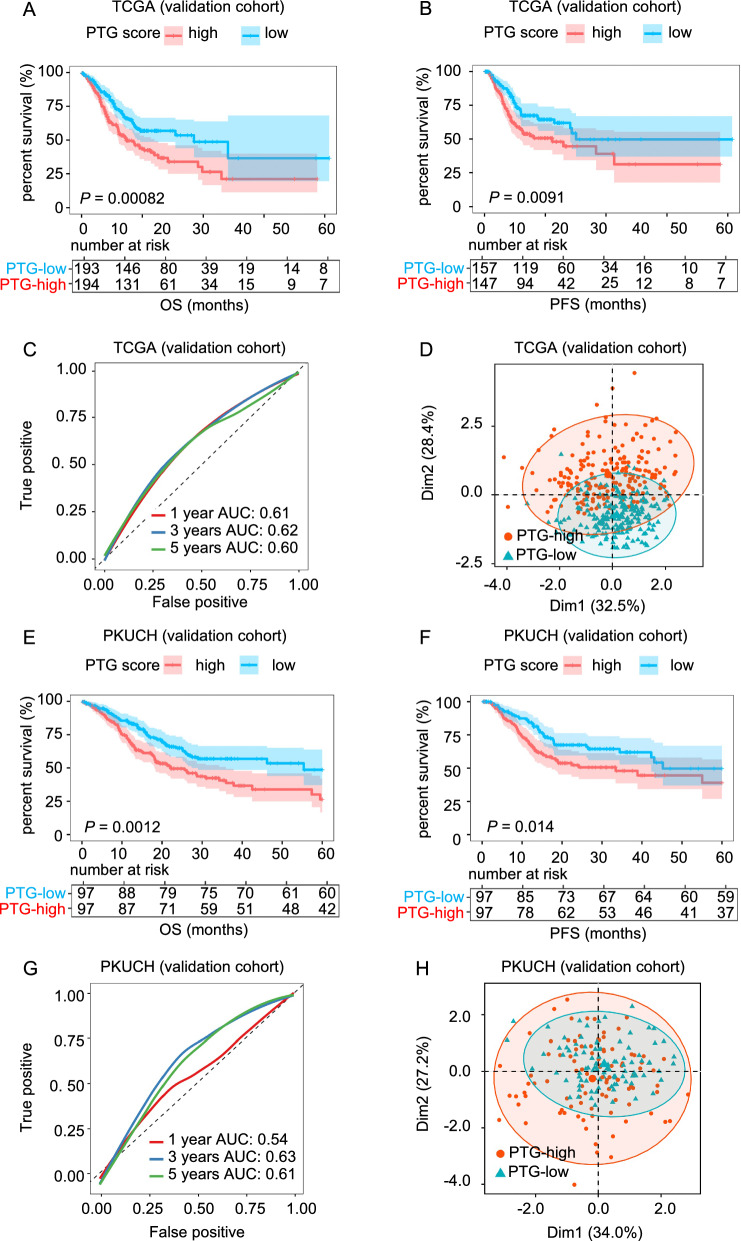
Validation of the PTG score in The Cancer Genome Atlas and Peking University Cancer Hospital cohorts. **A**, **B** Kaplan–Meier curves for the OS and PFS of GC patients in the low and high PTG score groups from The Cancer Genome Atlas (TCGA) cohort. **C** AUC of time-dependent ROC curves at 1, 3 and 5 years for the TCGA cohort. **D** PCA analysis showed different distribution patterns in the low and high PTG score groups from the TCGA cohort. The ellipse represents the 95% confidence interval. **E**, **F** Kaplan–Meier curves for the OS and PFS of GC patients in the low group and high PTG score groups from the Peking University Cancer Hospital (PKUCH) cohort. **G** AUC of time-dependent ROC curves at 1, 3 and 5 years from the PKUCH cohort. **H** PCA showed different distribution patterns in the low and high PTG score groups from the PKUCH cohort. The ellipse represents the 95% confidence interval

Next, we used the TCGA STAD and PKUCH cohorts to further validate the feasibility of PTG score for predicting GC prognosis. To maintain consistency with the training cohort, the cutoff values for the low- and high-risk groups were determined based on the median PTG scores. The results of prognostic analysis were consistent with those of the ACRG training cohort. Kaplan–Meier survival curves showed that OS and PFS were poorer in the high-risk group than in the low-risk group (Fig. 6A, B, E and F). In the TCGA cohort, the AUCs for 1-, 3-, and 5-year OS were 0.61, 0.62, and 0.60, respectively (Fig. 6C). In the PKUCH cohort, the AUCs for 1-, 3-, and 5-year OS were 0.54, 0.63, and 0.61, respectively (Fig. 6G). These relatively lower AUC values may be caused by lower transcriptome differences between the PTG-high and PTG-low groups, higher intra-group variation, and RNA-seq batch effects. To investigate the batch effect, we performed PCA by combining the training and the two validation cohorts. As shown in Additional file 1: Fig. S5A, there was a batch effect between the training and validation cohorts, although the PTG score was predictive in either cohort. PCA analysis based on PTG-related genes suggested that the PTG expression pattern difference between the high and low PTG score groups were compromised in the TCGA and PKUCH validation cohorts (Fig. 6D, H). Furthermore, we performed PCA of the whole transcriptome and calculated the Euclidean distances between every two samples in the training and validation sets. The results showed that, when considering the whole transcriptome in the PCA analysis, the expression pattern differences between the high and low PTG score groups in the validation cohorts became less obvious than in the training cohort, but were still detectable (Additional file 1: Fig. S6A–C). More importantly, the intragroup Euclidean distance distribution curves of the training and validation cohorts demonstrated increased intragroup variations within the high PTG score groups, with intragroup variations being especially large in the two validation cohorts. This result suggested higher levels of transcriptomic heterogeneity in tumors with a high PTG score, especially those from the validation cohorts (Additional file 1: Fig. S6D–F).

Although the prognostic predictive value of the PTG score was effectively validated by ROC analysis in patients with GC from both the training and validation cohorts, tumor transcriptomic heterogeneity and RNA-seq batch effects still posed limitations to this study. To further test the robustness of the PTG score at predicting GC prognosis, prospective studies with larger and more representative cohorts are needed.

### The PTG score correlates with the immune cell infiltration landscape

**Fig. 7:**
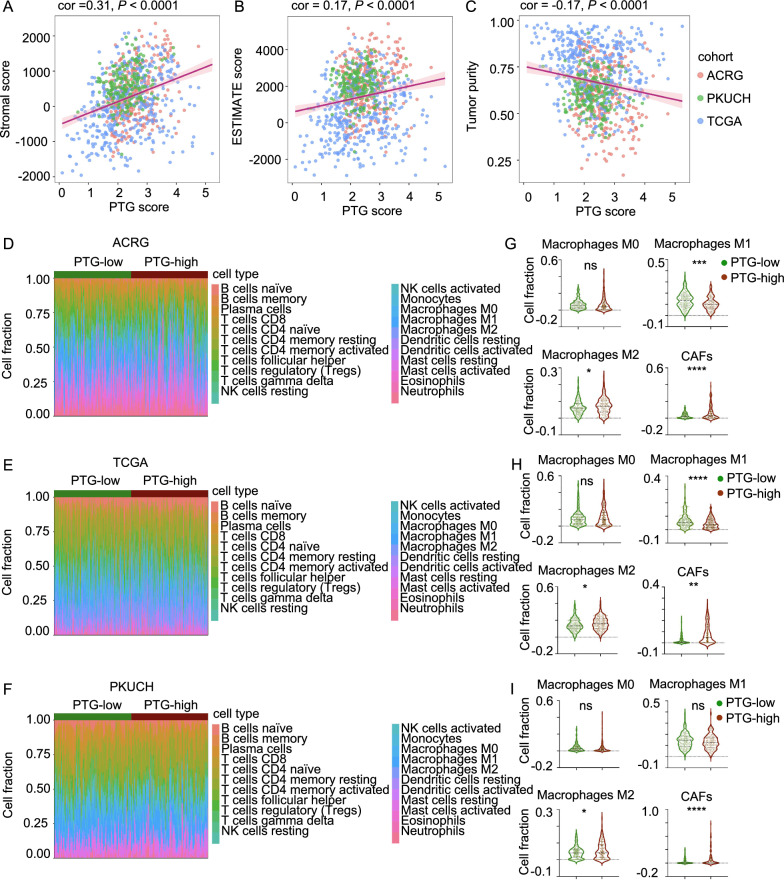
The PTG score correlates with the immune cell infiltration pattern. **A**–**C** Association between the PTG signature and stromal score, ESTIMATE score, and tumor purity. **D**–**F** Stacked histogram of the ratio of 22 immune cell types in GC patients in the low- or high PTG score groups from the ACRG, TCGA, and PKUCH cohorts. **G**–**I** The different distributions of immune cells in the low and high PTG score groups from the ACRG, TCAG, and PKUCH cohorts

The ECM of tumors plays a key role in immune cell infiltration [17] and contributes to tumor ECM remodeling [10, 13]. Next, we performed immune cell infiltration estimation analysis. First, we calculated the stromal score, ESTIMATE score, and tumor purity using ESTIMATE [31]. The results showed a positive correlation between the PTG score and stromal score and the ESTIMATE score, and a negative correlation between the PTG score and tumor purity (Fig. 7A–C). We analyzed 22 types of immune cell infiltration landscapes using the CIBERSORTx algorithm [30]. The patients in the three cohorts were divided into two groups based on the median PTG score. The 22 immune cell proportion plots indicated different immune microenvironmental patterns (Fig. 7D–F). TIMER has previously been used to estimate the degree of immune cell infiltration [32]. Of all immune cells analyzed, macrophages and CAFs showed the most significant differential infiltration. Specifically, M0 macrophage infiltration was similar in the low PTG score and high PTG score groups, classically activated macrophage (M1) infiltration was lower in the high PTG score group, alternatively activated macrophage (M2) infiltration was higher in the high PTG score group, and the proportion of CAFs was higher in the high PTG score group (Fig. 7G–I). Since M1 macrophages are regarded as having an antitumor phenotype, and M2 macrophages have been reported to contribute to immune suppression and tumor cell metastasis [33–36], we inferred that, as the PTG score increases, macrophages are more inclined to be M2-polarized, leading to a more friendly microenvironment for tumor cells to escape immune surveillance. Additionally, CAFs showed a higher proportion in patients in the high PTG score group. CAFs are known for their key roles in the TME [37]. They act as tumor-promoting cells by remodeling the ECM; producing growth factors; and regulating tumor cell progression, metabolism, and angiogenesis [38–40]. Based on the above results, we propose that a high PTG score indicates a protumor immune microenvironment, which leads to a poor prognosis in patients with GC.

### Correlation between the PTG score and GC clinical characteristics

**Fig. 8:**
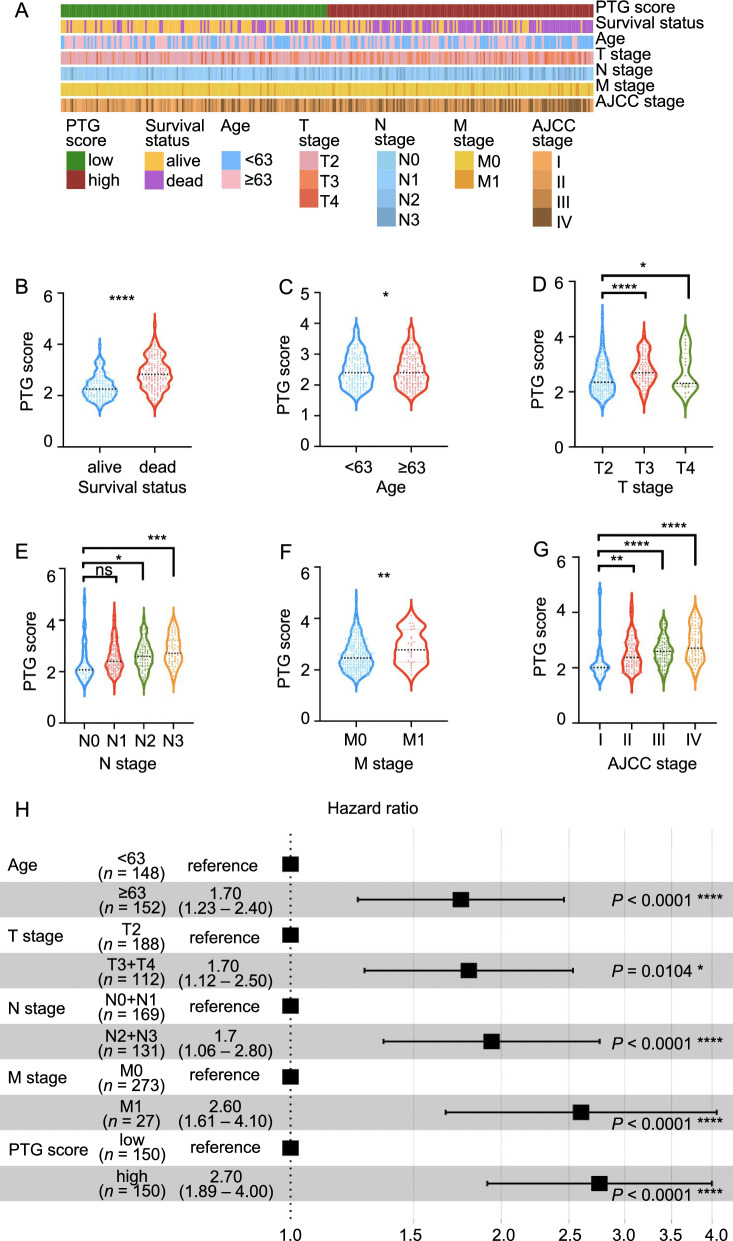
Correlations between PTG scores and clinical characteristics in GC. **A** Heatmap of the clinicopathological characteristics and PTG scores. **B**–**G** The PTG scores in different groups classified based on clinical characteristics from the ACRG, TCGA, and PKUCH sets. **H** Multivariate Cox regression analyses of OS in the ACRG cohort. *P < 0.05, **P < 0.01, ***P < 0.001, ****P < 0.0001

Next, we investigated the clinical characteristics, including 5-year survival, age, T stage, N stage, M stage, and American Joint Committee on Cancer (AJCC) pTNM stage, in the low and high PTG score groups (Fig. 8A). There were significant differences in the distributions of clinical characteristics between the two groups. Consistent with the above findings, the 5-year survival rate was significantly lower in the high PTG score group (Fig. 8B). Moreover, GC patients aged greater than 63 years exhibited higher PTG scores (Fig. 8C). Additionally, a high PTG score was correlated with a more advanced TNM stage (Fig. 8D–G). These results indicate that the PTG score is associated with a higher risk of poor clinical outcomes. Next, to explore whether the PTG score could be used as an independent predictor of GC prognosis, we performed univariate and multivariate Cox regression analyses. The results showed that, together with age, T stage, N stage, and M stage, the PTG score was an independent prognostic factor for OS (Fig. 8H; Additional file 2: Table S4).

### Construction and validation of an integrative nomogram

**Fig. 9:**
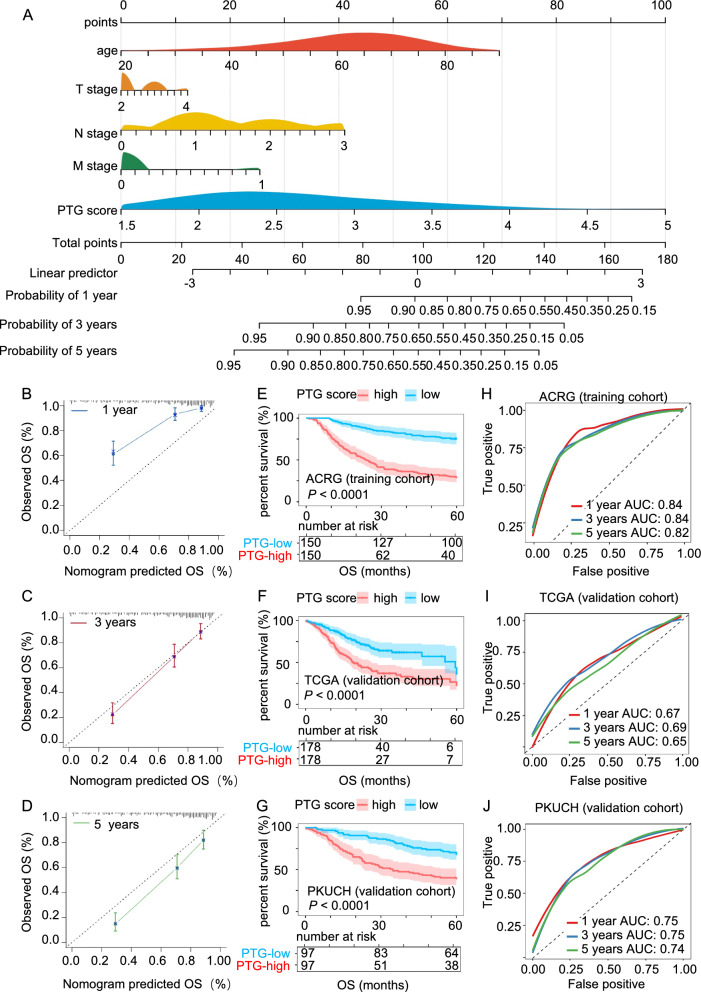
Nomogram combining AJCC stage and PTG score to predict the OS of GC patients. **A** Nomogram integrating the PTG score, age, T stage, N stage, and M stage for predicting the 1-, 3-, and 5-year OS of GC patients in the training set. **B**–**D** Calibration curves of the nomogram for predicting the 1-, 3- and 5-year OS in the ACRG, TCGA, and PKUCH cohorts. **E**–**G** Kaplan–Meier survival plots of OS in the ACRG, TCGA, and PKUCH cohorts. **H**–**J** ROC curves for predicting the 1-, 3-, and 5-year OS in the ACRG, TCGA, and PKUCH cohorts

As the independent prognostic analyses described above revealed that age, T stage, N stage, M stage, and PTG score were independent prognostic factors for GC, we established an integrative nomogram to predict the 1-, 3-, and 5-year OS of patients with GC (Fig. 9A). The calibration charts illustrated that the OS probability predicted by the nomogram approximated the actual probability well, and the 3-year OS prediction agreed best with the actual OS (Fig. 9B–D). We analyzed the relationship between survival status and nomogram scores in both the training and validation sets. Kaplan–Meier survival analysis showed that GC patients with higher nomogram scores had poorer OS in the ACRG (*P* < 0.001), TCGA (*P* < 0.0001), and PKUCH cohorts (*P* < 0.0001; Fig. 9E–G). In the training set, the AUCs for OS at 1, 3, and 5 years, according to the nomogram model, indicated high prognostic validity. Specifically, the AUC values of the ACRG cohort for 1-, 3-, and 5-year OS were 0.843, 0.837, and 0.824, respectively (Fig. 9H). In the TCGA validation set, the AUC values for 1-, 3-, and 5-year OS were 0.667, 0.694, and 0.649, respectively (Fig. 9I). In the PKUCH validation set, the AUC values for 1-, 3-, and 5-year OS were 0.753, 0.745, and 0.739, respectively (Fig. 9J). These results illustrated the effectiveness of the nomogram. Furthermore, to compare the predictive potential of the nomogram and other clinical characteristics, we constructed ROC curves of the nomogram, age, T stage, N stage, M stage, and pTNM stage at 3 years OS. In the training set, the AUC value of the nomogram was 0.837, which was higher than the AUC values for age (0.551), T stage (0.633), N stage (0.717), M stage (0.578), and pTNM stage (0.744; Additional file 1: Fig. S7A–B). In the validation set, the conclusion was similar to that of the training set. Specifically, in the TCGA validation set, the AUC values of the nomogram, age, T stage, N stage, M stage, and pTNM stage were 0.694, 0.578, 0.559, 0.624, 0.527, and 0.608, respectively (Additional file 1: Fig. S7C–D). In the PKUCH validation set, the AUC values of the nomogram, age, T stage, N stage, M stage, and pTNM stage were 0.745, 0.543, 0.551,0.736, 0.540, and 0.695, respectively (Additional file 1: Fig. S7E–F). These results showed that our nomogram combining PTG score, age, T stage, N stage, and M stage was more effective than age or TNM stage alone at predicting GC prognosis.

## Discussion

PDTX models have been widely used in clinical research [41]. To create a PDTX model, cancer tissue was cut into small pieces of approximately 3 mm^3^, and then implanted subcutaneously in mice according to published methods [22]. Thus, PDTX models not only provide an in vivo microenvironment for investigating the tumorigenic potential of tumor cells, but also help maintain the interaction between tumor cells and other cells within the TME. Therefore, in addition to their application in drug screening, PDTX models can act as biological-function-testing models to measure the tumorigenic ability of tumor tissues. We noticed a significant variation in the tumorigenicity of tumor tissues from 122 patients with GC, even for those in the same age group and pathological stage. Further analysis revealed a correlation between tumorigenicity and GC prognosis. Notably, GC at the T1 stage show only a 25% tumorigenesis rate. This suggests that patients with T1 stage GC might not be suitable for PDTX model construction. However, owing to the limited number of T1 samples (four samples in this study), the low tumorigenesis rate was not statistically significant. Whether GC tissues at the T1 stage possess unique molecular characteristics that hinder tumorigenesis requires further investigation.

To investigate the mechanism underlying the prognosis predicting potential of PDTX tumorigenicity, we performed RNA-seq analysis of PDTX-originating tumor tissues and their corresponding normal tissues from 14 patients in the tumorigenesis group and five in the non-tumorigenesis group. By profiling the transcriptomes of these samples, we discovered that multiple ECM-related signaling pathway gene sets were enriched in the tumorigenesis group. This suggests that the ECM may play key roles in tumorigenesis in PDTX models. In addition, ECM-related prognostic signatures have been identified across multiple solid tumor types, including GC [42–46], non-small cell lung cancer, pancreatic ductal adenocarcinoma, ovarian cancer, hepatocellular carcinoma, and triple-negative breast cancer [47, 48]. However, these analyses focused only on prognosis and lacked animal models to confirm the in vivo role of the ECM signatures in tumor tissues.

Based on the ECM-related genes discovered in the tumorigenesis group, we constructed a robust GC prediction model using LASSO Cox regression analysis, namely, the PTG score model. This model had AUC values of 0.72, 0.76, and 0.75 for 1-, 3-, and 5-year OS rates, respectively. The predictive ability of the PTG score was validated using the TCGA STAD dataset and our PKUCH cohort. Furthermore, GC patients with different PTG scores showed heterogeneous pathological characteristics and immune landscapes. In the high PTG score group, macrophages were more inclined to be M2 polarized, which has been reported to be a tumor-promoting phenotype. Additionally, the proportion of patients with CAF infiltration was higher in the high PTG score group.

The ECM consists of a variety of macromolecules that maintain the architecture, integrity, development, and homeostasis of normal tissues [49, 51]. The dynamic reciprocity between the ECM and the cells within it has long been observed [52]. The loss of correct ECM organization and homeostasis is a hallmark of solid tumors, in which both tumor and non-malignant stromal cells contribute to, and in turn are affected by, the deposition and remodeling of the ECM [16]. Biochemical and biomechanical changes in the ECM result in dysregulation of intracellular signaling in tumor cells and the promotion of proliferation, survival, and resistance to chemotherapy [53]. Conversely, to induce surrounding non-malignant cells to support tumor cell migration and invasion, tumor cells often subvert nearby stromal cells to remodel the ECM, constituting a crosstalk pattern that differs from the pattern in non-malignant conditions [17]. In addition, CAFs can remodel the ECM by degrading normal ECM components; secreting multiple matrix proteins; producing MMPs, including MMP-1 and MMP-3; increasing ECM stiffness; and facilitating tumor progression [54–58]. The remodeled ECM then promotes CAF activation [59]. Moreover, there is an interaction between tumor-associated macrophages (TAMs) and CAFs [60]. For instance, CAFs promote monocyte migration and polarization into the M2 phenotype in breast and prostate carcinomas [61–64]. Reciprocally, TAMs in the M2 polarization state facilitate CAF activation and progression [65].

Therefore, we suggest that, in our study, the ECM-related signaling pathways were dysregulated in patients whose originating tumor tissue successfully formed subcutaneous tumors in the PDTX model. This dysregulation results in changes to tumor intracellular signaling and immune cell infiltration, especially by CAFs and TAMs. In turn, the tumor cells and infiltrated immune cells promote remodeling of the ECM, forming a positive feedback loop and facilitating primary tumor development and progression in patients and tumorigenesis in mice. Thus, by post-surgery pathological diagnosis, we could not identify patients with a highly remodeled ECM. However, although the GC tissue was surgically removed, the ECM of the highly remodeled tumor may have already released numerous tumor cells for metastasis before surgery. Previous studies have shown that a large fraction of disseminated tumor cells are solitary, mitotically quiescent cells that are often referred to as dormant cells [66]. Dormant tumor cells can re-activate to re-enter the cell cycle after months or years, and rapidly develop lesions [67]. This may be the underlying mechanism of the poor prognosis in the PDTX tumorigenesis group of GC patients. Importantly, the remodeled ECM is a potential target for inhibiting GC metastasis and recurrence. To target the remodeled ECM, the use of multi-vesicular vesicles is a promising alternative to existing therapeutics [68]. Specifically, extracellular vesicles can be applied in drug delivery and release [69], with the potential to target cancer cells and organelles, including the mitochondria. The high plasticity and load capacity of engineered extracellular vesicles make it possible to achieve various combinations of cancer treatment methods [70]. Nanomaterials have been widely studied as vectors to improve drug delivery in cancer therapy [71]. For instance, in lung cancer, by encapsulating doxorubicin in superparamagnetic iron oxide nanoparticles, the drug can be released in a controlled manner, which may become a powerful chemopreventive and chemotherapeutic system for patients [72]. However, further studies are required to confirm these hypotheses in patients with GC.

Finally, we integrated the PTG score, age, and pathological stage information to construct an effective nomogram for predicting GC prognosis. The AUC values for 1-, 3-, and 5-year OS in the training sets were 0.843, 0.837, and 0.824, respectively. The prognosis-predicting ability of the nomogram was also validated in the TCGA STAD and PKUCH cohorts. In the TCGA validation set, the AUC values for 1-, 3-, and 5-year OS were 0.667, 0.694, and 0.649, respectively. In the PKUCH validation set, the AUC values for 1-, 3-, and 5-year OS were 0.753, 0.745, and 0.739, respectively. We further compared the prognostic predictive ability of the nomogram with the predictive ability of age and pathological characteristics. The results showed that our integrative nomogram combining the PTG score, age, T stage, N stage, and M stage was more effective than age or TNM stage alone at predicting GC prognosis.

Although our study provides new insights into prognostic prediction and targeted therapy for GC, it has several limitations. First, owing to the limited number of GC samples used for RNA-seq, the prognostic PTG score model was established and validated using retrospective datasets. Large-scale prospective clinical cohorts are required to test the robustness of the model. Second, we applied bulk RNA-seq to GC tissues and their corresponding normal tissues. Further studies should include single-cell RNA-seq to validate the expression signatures of stromal and immune cells in the ECM. Finally, in vitro and in vivo assays are needed to validate the correlation between the PTG score and immune cell infiltration, which will provide new insights into GC prognosis prediction and individualized therapy.

## Conclusions

In the present study, we used PDTX models in 122 GC cases and found that PDTX tumorigenicity was an independent prognostic factor. By investigating the transcriptome of PDTX-originating tumor cells, we found that ECM-related genes were strongly associated with tumorigenicity. Thus, we established a feasible model, named the PTG score, to predict GC prognosis. The predictive ability of the model was more robust when combined with patient age and pathological TNM stage. In addition, we revealed the relationship between PTG score and immune cell infiltration in tumors. Specifically, a high PTG score was significantly associated with CAF infiltration and macrophage M2 polarization, which may be promising targets for individual GC treatment. Overall, together with patient age and pathological TNM stage, the PTG score may be used as an effective tool for predicting GC prognosis.

### Supplementary Information

**Additional file 1: Figure S1**. Representative HE (hematoxylin-eosin) staining of GC tissue from non-tumorigenesis group. (A-B) Representative HE staining of GC tissue from non-tumorigenesis group. **Figure S2**. Survival analysis of patients for PDTX models. (A-P) Kaplan-Meier curves for OS and DFS of 122 GC patients grouped by clinical characteristics. **Figure S3**. (A) The protein-protein interaction (PPI) evidence among coding DEGs in non-tumorigenesis group. (B) GO enrichment bar plots of 36 DEGs for biological process (green), cellular component (red), and molecular function (blue). **Figure S4**. (A) The Pearson correlation analysis among 116 candidate prognostic genes. **Figure S5**. PCA analysis of training cohort and validation cohorts. (A) PCA analysis showed batch effect among ACRG training set and both validation sets. Ellipse represents 95% confidence interval. **Figure S6**. PCA analysis and Euclidean distances distribution of PTG score-low groups and PTG-score high groups in training cohort and validation cohorts. (A) PCA analysis showed different gene expression distribution patterns in the PTG score-low group and PTG score-high group from ACRG training cohort. Ellipse represents 95% confidence interval. (B-C) PCA analysis showed different gene expression distribution patterns in the PTG score-low groups and PTG score-high groups from TCGA cohort and PKUCH cohort. Ellipses represents 95% confidence interval. (D-F) Distribution plot of Euclidean distances between every two samples in the PTG score-low group and PTG score-high group from ACRG, TCGA and PKUCH cohorts. **Figure S7**. Comparison of AUCs of nomogram and age/TNM stages. (A) AUCs of the nomogram, age, T stage, N stage and M stage to predict OS at 1 year using ACRG cohort. (B) AUCs of the nomogram, age and TNM stage to predict OS at 1 year using ACRG cohort. (C) AUCs of the nomogram, age, T stage, N stage and M stage to predict OS at 1 year using TCGA cohort. (D) AUCs of the nomogram, age and TNM stage to predict OS at 1 year using TCGA cohort. (E) AUCs of the nomogram, age, T stage, N stage and M stage to predict OS at 1 year using PKUCH cohort. (F) AUCs of the nomogram, age and TNM stage to predict OS at 1 year using PKUCH cohort.

**Additional file 2: Table S1**. Cox regression analysis of overall survival and PDTX tumorigenicity together with clinical characteristics. **Table S2**. Clinical characteristics of 14 tumorigenesis GC patients for RNA-seq. **Table S3**. Clinical characteristics of 5 non-tumorigenesis tumorigenesis GC patients for RNA-seq. **Table S4**. Cox regression analysis of overall survival and PTG score together with clinical characteristics.

## Data Availability

The RNA-seq raw data (fastq files) for the 19 PDTX-originating GC tissues and their corresponding normal tissues generated and analyzed during the current study are available from the NGDC repository (https://ngdc.cncb.ac.cn/, accession ID: HRA004403). The gene expression dataset of the ACRG cohort analyzed in the current study is available at the GEO database repository, https://www.ncbi.nlm.nih.gov/geo/query/acc.cgi?acc=GSE62254 [23]. The gene expression dataset of TCGA cohort analyzed in the current study is available at the UCSC repository, https://xenabrowser.net/datapages/. The gene expression dataset of the PKUCH cohort analyzed in the current study is available from the corresponding author upon reasonable request [24].

## Abbreviations

GC: Gastric cancer
PDTX: Patient-derived tumor xenograft
ECM: Extracellular matrix
CAF: Cancer-associated fibroblasts
TME: Tumor microenvironment
IHC: Immunohistochemistry
FISH: Fluorescence in situ hybridization
NCCN: National Comprehensive Cancer Network
CSCO: Chinese Society of Clinical Oncology
HER2: Human epidermal growth factor receptor 2
PD-L1: Programmed death protein-1
MSI: Microsatellite instability
MMR: Mismatch repair
NTRK: Neurotrophic tropomysin-related kinase
EBV: Epstein-Barr virus
PKUCH: Peking University Cancer Hospital
ACRG: Asian Cancer Research Group
TCGA: The Cancer Genome Atlas
STAD: Stomach adenocarcinoma
UCSC: University of California Santa Cruz
OS: Overall survival
PFS: Progression free survival
GEO: Gene expression omnibus
RNA-seq: RNA-sequencing
DEG: Differentially expressed gene
GO: Gene ontology
LASSO: Least absolute shrinkage and selection operator
PTG: PDTX tumorigenicity-related genes
ESTIMATE: Estimation of stromal and immune cells in malignant tumors using expression data
Nod/Scid: Non-obese diabetes/severe combined immune-deficiency
POT: PDTX originating tumor
CC: Cellular component
BP: Biological process
MF: Molecular function
ROC: Receiver-operating characteristic
AUC: Area under the ROC curve
PCA: Principal components analysis
AJCC: American Joint Committee on Cancer
HE: Hematoxylin–eosin
NGDC: National Genomics Data Center
lncRNA: Long non-coding RNA
TAM: Tumor-associated macrophage
