# Supplementary figures and images for: Transcriptome profiling of patient-derived tumor xenografts suggests novel extracellular matrix-related signatures for gastric cancer prognosis prediction
Source: J Transl Med. 2023 Sep 19;21:638. doi: 10.1186/s12967-023-04473-0 (PMC10510236; doi:10.1186/s12967-023-04473-0)

Figure S1

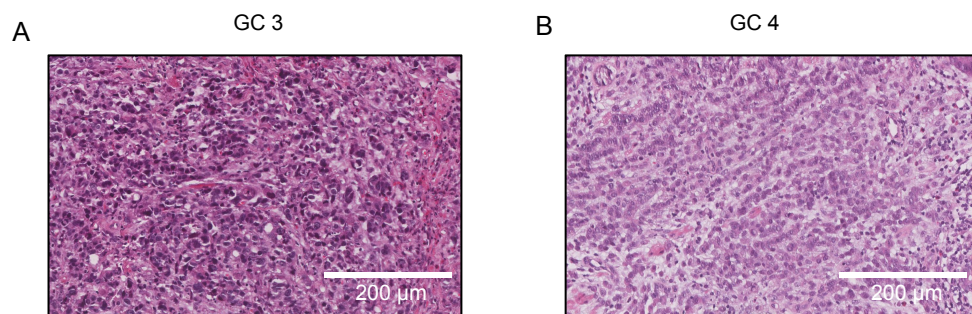

Figure S2

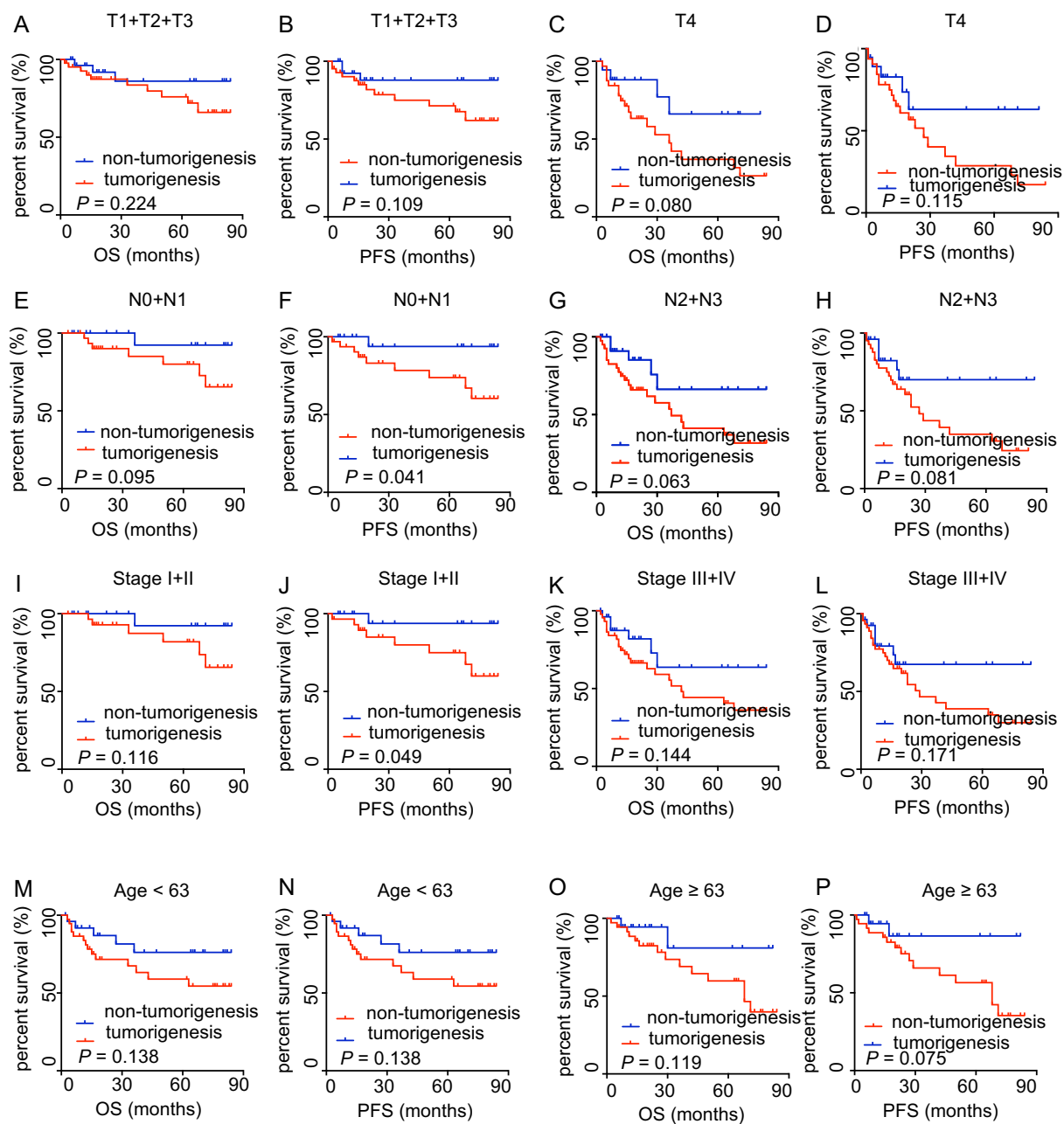

Figure S3

A

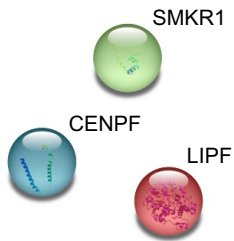

B

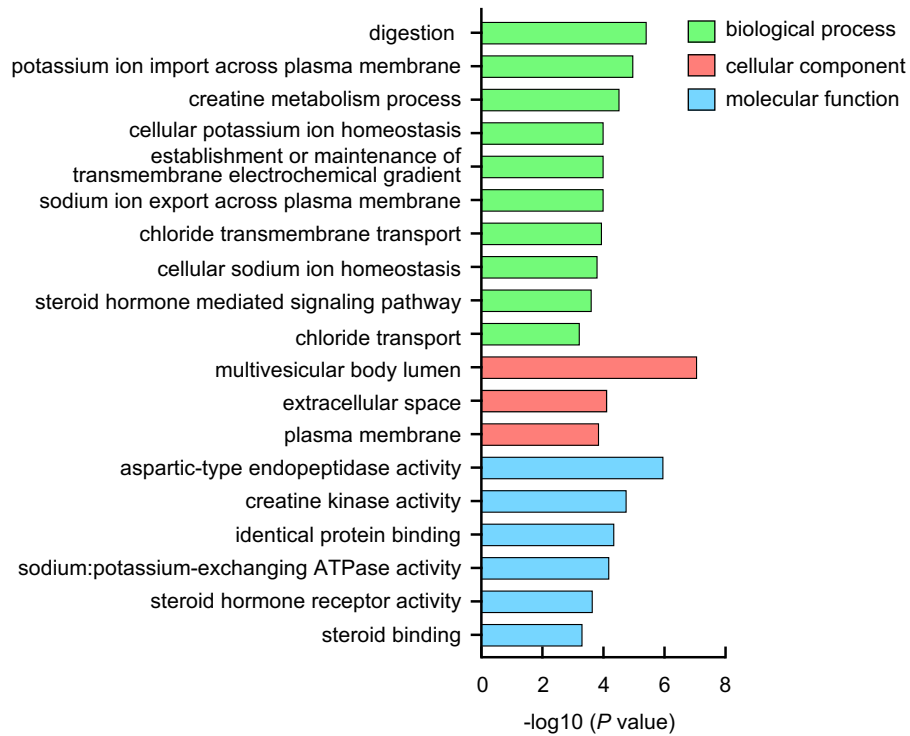

A

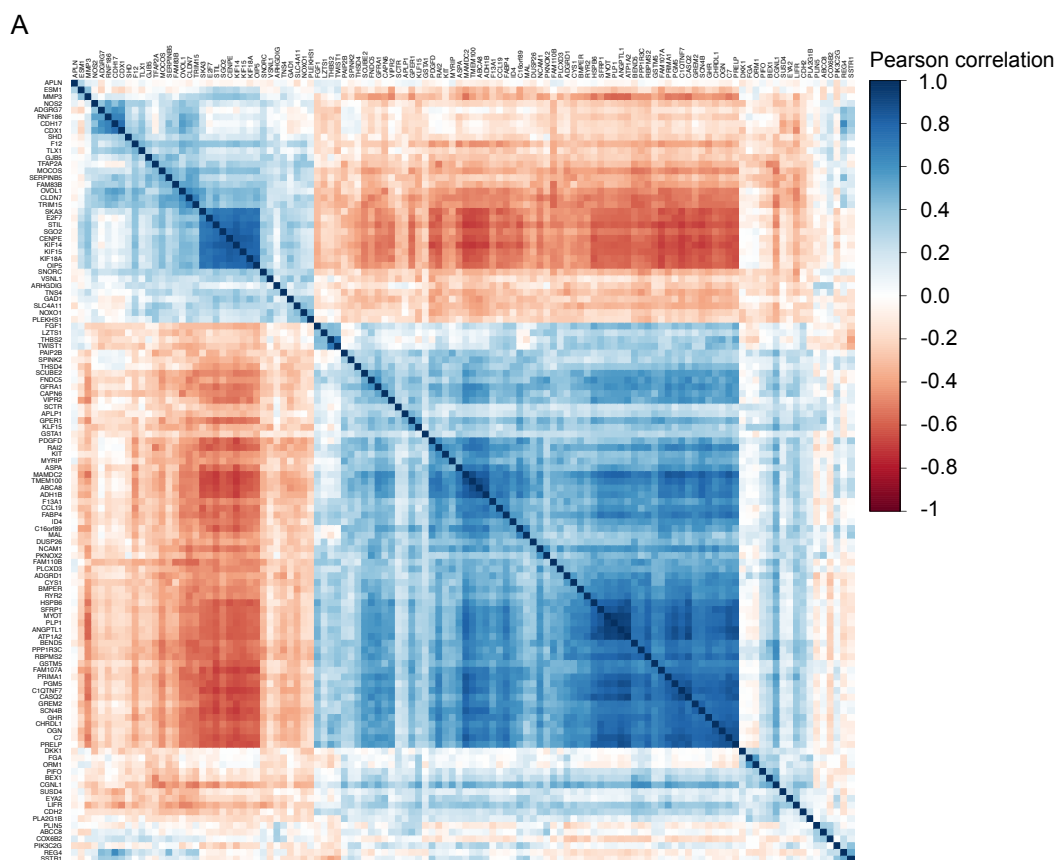

Figure S5

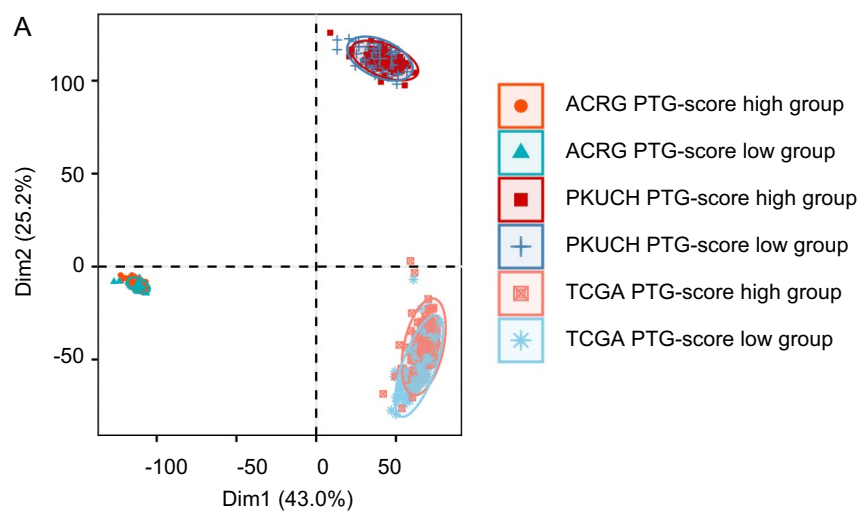

Figure S6

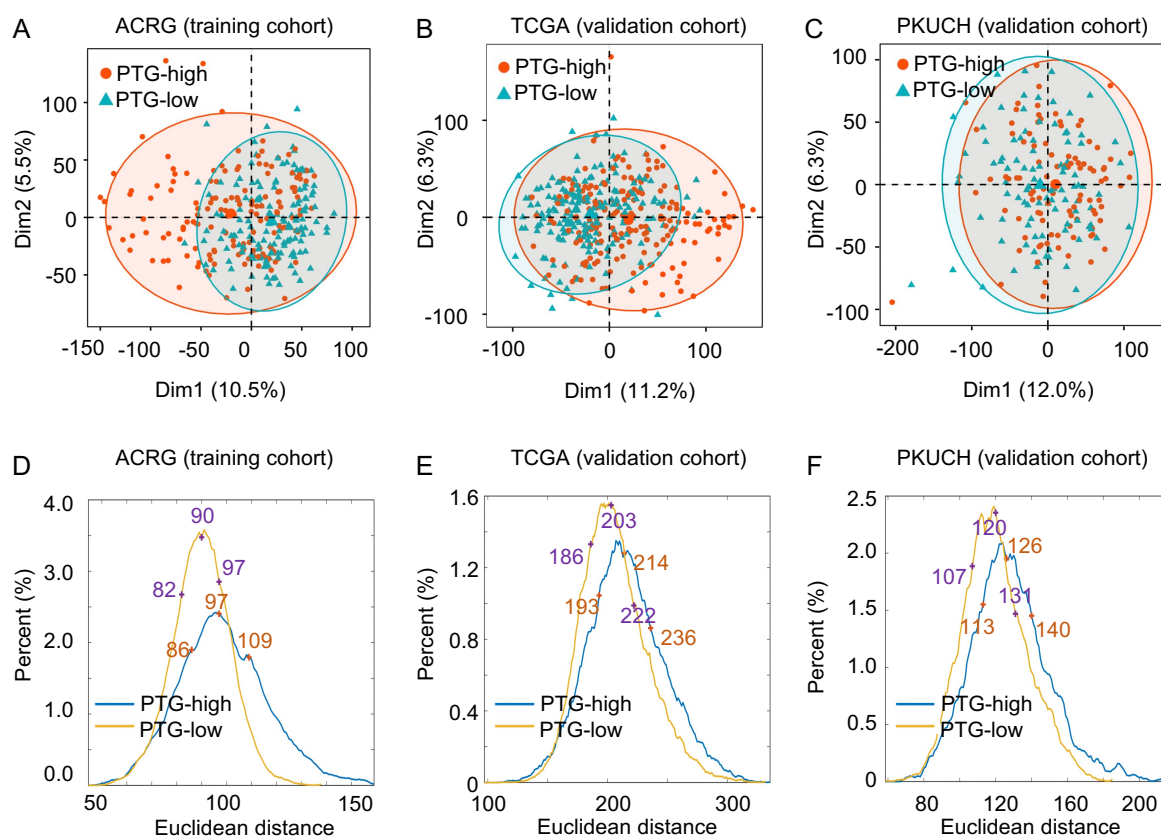

Figure S7

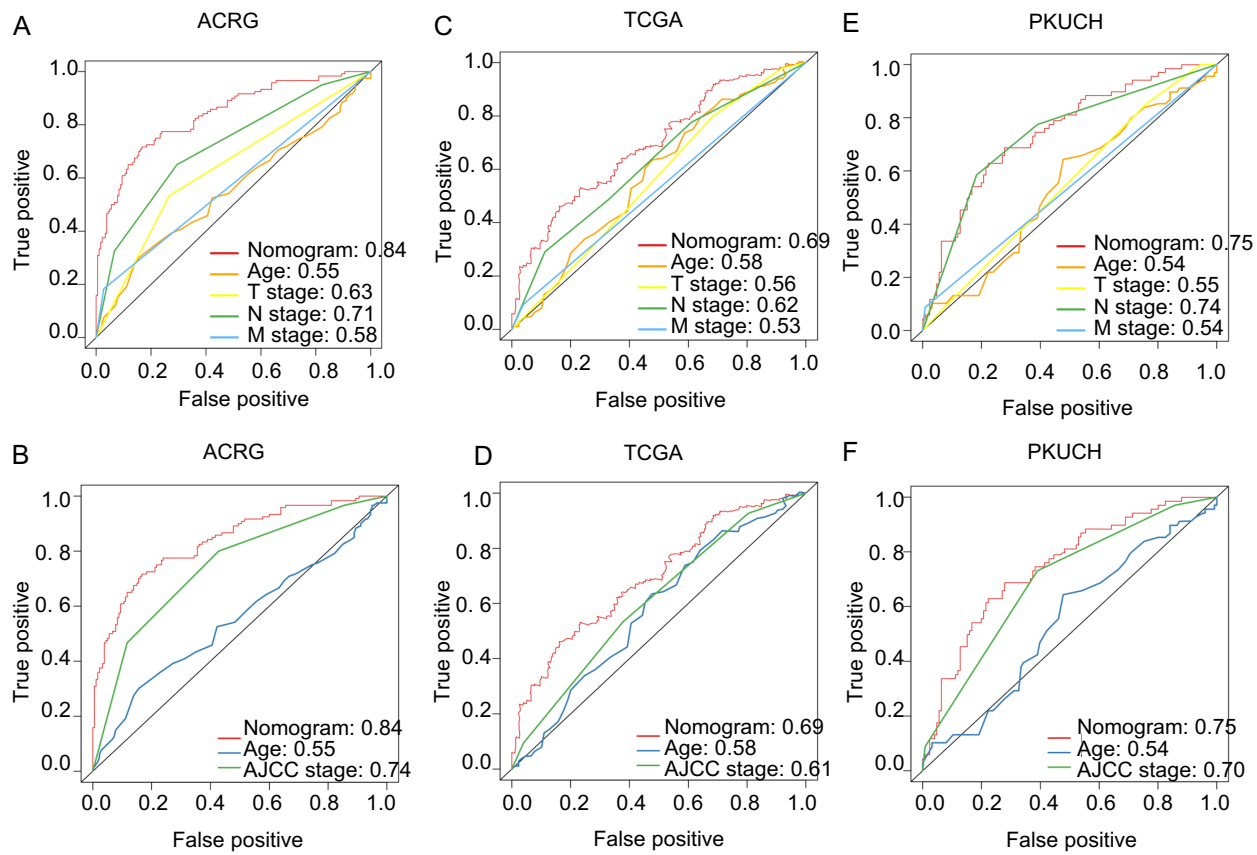

Supplement: Supplementary file 1 — Additional file 1: Figure S1. Representative HE (hematoxylin-eosin) staining of GC tissue from non-tumorigenesis group. (A-B) Representative HE staining of GC tissue from non-tumorigenesis group. Figure S2. Survival analysis of patients for PDTX models. (A-P) Kaplan-Meier curves for OS and DFS of 122 GC patients grouped by clinical characteristics. Figure S3. (A) The protein-protein interaction (PPI) evidence among coding DEGs in non-tumorigenesis group. (B) GO enrichment bar plots of 36 DEGs for biological process (green), cellular component (red), and molecular function (blue). Figure S4. (A) The Pearson correlation analysis among 116 candidate prognostic genes. Figure S5. PCA analysis of training cohort and validation cohorts. (A) PCA analysis showed batch effect among ACRG training set and both validation sets. Ellipse represents 95% confidence interval. Figure S6. PCA analysis and Euclidean distances distribution of PTG score-low groups and PTG-score high groups in training cohort and validation cohorts. (A) PCA analysis showed different gene expression distribution patterns in the PTG score-low group and PTG score-high group from ACRG training cohort. Ellipse represents 95% confidence interval. (B-C) PCA analysis showed different gene expression distribution patterns in the PTG score-low groups and PTG score-high groups from TCGA cohort and PKUCH cohort. Ellipses represents 95% confidence interval. (D-F) Distribution plot of Euclidean distances between every two samples in the PTG score-low group and PTG score-high group from ACRG, TCGA and PKUCH cohorts. Figure S7. Comparison of AUCs of nomogram and age/TNM stages. (A) AUCs of the nomogram, age, T stage, N stage and M stage to predict OS at 1 year using ACRG cohort. (B) AUCs of the nomogram, age and TNM stage to predict OS at 1 year using ACRG cohort. (C) AUCs of the nomogram, age, T stage, N stage and M stage to predict OS at 1 year using TCGA cohort. (D) AUCs of the nomogram, age and TNM stage to predict OS [file 12967_2023_4473_MOESM1_ESM.pdf]
