# Supplementary material for: Transcriptome profiling of patient-derived tumor xenografts suggests novel extracellular matrix-related signatures for gastric cancer prognosis prediction
Source: J Transl Med. 2023 Sep 19;21:638. doi: 10.1186/s12967-023-04473-0 (PMC10510236; doi:10.1186/s12967-023-04473-0)
Supplement: Supplementary file 2 — Additional file 2: Table S1. Cox regression analysis of overall survival and PDTX tumorigenicity together with clinical characteristics. Table S2. Clinical characteristics of 14 tumorigenesis GC patients for RNA-seq. Table S3. Clinical characteristics of 5 non-tumorigenesis tumorigenesis GC patients for RNA-seq. Table S4. Cox regression analysis of overall survival and PTG score together with clinical characteristics. [file 12967_2023_4473_MOESM2_ESM.docx]

Table S1. Cox regression analysis of overall survival and PDTX tumorigenicity together with clinical characteristics

| **Variable** | **Univariate analysis** | | | |
| --- | --- | --- | --- | --- |
|  | **N** | **HR (95%CI)** | ***P* value** | **Sig** |
| **Tumorigenesis** |  |  |  |  |
| No | 48 | ref |  |  |
| Yes | 74 | 2.51 | 0.030 | * |
|  |  | (1.10-5.75) |  |  |
| **Age** | >63 | ref | 0.930 |  |
|  | ≤63 | 1.03 |  |  |
|  |  | (0.53-2.01) |  |  |
| **Gender** |  |  |  |  |
| Female | 37 | ref |  |  |
| Male | 85 | 1.23 | 0.590 |  |
|  |  | (0.58-2.63) |  |  |
| **T stage** |  |  |  |  |
| T1+T2+T3 | 71 | ref |  |  |
| T4 | 51 | 3.165 | 0.001 | *** |
|  |  | (1.59-6.30) |  |  |
| **N stage** |  |  |  |  |
| N0+N1 | 54 | ref |  |  |
| N2+N3 | 66 | 4.07 | <0.001 | *** |
|  |  | (1.83-9.04) |  |  |
| **M stage** |  |  |  |  |
| M0 | 108 | ref |  |  |
| M1 | 14 | 2.18 | 0.108 |  |
|  |  | (0.84-5.65) |  |  |
| **AJCC stage** |  |  |  |  |
| I+II | 50 | ref |  |  |
| III+IV | 72 | 4.31 | <0.001 | *** |
|  |  | (1.87-9.93) |  |  |
| **Lauren type** |  |  |  |  |
| intestinal | 38 | ref |  |  |
| diffuse | 21 | 2.30 | 0.151 |  |
|  |  | (0.74-7.14) |  |  |
| mixed | 45 | 1.66 | 0.305 |  |
|  |  | (0.63-4.37) |  |  |

* *P*<0.05, ** *P*<0.01, *** *P*<0.001

Table S2. Clinical characteristics of 14 tumorigenesis GC patients for RNA-seq

| **Tumorigenesis group** | |
| --- | --- |
| **Character** | **N (%)** |
| **Gender** |  |
| Male | 10 (74.1) |
| Female | 4 (28.6) |
| **Age** |  |
| Male median age [range] | 63.5 [45-78] |
| Female median age [range] | 71.5 (65-78) |
| **Tumor stage** |  |
| II | 7 (50.0) |
| III | 5 (35.7) |
| IV | 2 (14.3) |
| **T stage** |  |
| T2 | 1 (7.1) |
| T3 | 9 (64.3) |
| T4 | 4 (28.6) |
| **N stage (n=120)** |  |
| N0 | 4 (28.6) |
| N1 | 4 (28.6) |
| N2 | 3 (21.4) |
| N3 | 3 (21.4) |
| **M stage** |  |
| M0 | 12 (85.7) |
| M1 | 2 (14.3) |
| **Lauren classification (n=121)** |  |
| Intestinal | 5 (35.7) |
| Diffuse | 1 (7.1) |
| Mixed | 8 (57.1) |

Table S3. Clinical characteristics of 5 non-tumorigenesis tumorigenesis GC patients for RNA-seq

| **Non-tumorigenesis group** | |
| --- | --- |
| **Character** | **N (%)** |
| **Gender** |  |
| Male | 3 (60.0) |
| Female | 2 (40.0) |
| **Age** |  |
| Male median age [range] | 44 [35-63] |
| Female median age [range] | 47.5 [35-60] |
| **Tumor stage** |  |
| III | 3 (60.0) |
| IV | 2 (40.0) |
| **T stage** |  |
| T3 | 1 (20.0) |
| T4 | 4 (80.0) |
| **N stage (n=120)** |  |
| N1 | 1 (20.0) |
| N3 | 4 (80.0) |
| **M stage** |  |
| M0 | 3 (60.0) |
| M1 | 2 (40.0) |
| **Lauren classification (n=121)** |  |
| Intestinal | 2 (40.0) |
| Diffuse | 1 (20.0) |
| Mixed | 2 (40.0) |

Table S4. Cox regression analysis of overall survival and PTG score together with clinical characteristics

| **Variable** | **Univariate analysis** | | | |
| --- | --- | --- | --- | --- |
|  | **N** | **HR (95%CI)** | ***P* value** | **Sig** |
| **Age** |  |  |  |  |
| ≤63 | 148 | ref |  |  |
| >63 | 152 | 1.28 | 0.100 |  |
|  |  | (0.92-1.77) |  |  |
| **T_stage** |  |  |  |  |
| T2 | 188 | ref |  |  |
| T4+T3 | 112 | 2.46 | <0.0001 | **** |
|  |  | (1.77-3.42) |  |  |
| **N_stage** |  |  |  |  |
| N0+N1 | 169 | ref |  |  |
| N2+N3 | 131 | 2.80 | <0.0001 | **** |
|  |  | (2.00-3.91) |  |  |
| **M_stage** |  |  |  |  |
| M0 | 273 | ref |  |  |
| M1 | 27 | 3.92 | <0.0001 | **** |
|  |  | (2.53-6.07) |  |  |
| **AJCC_stage** |  |  |  |  |
| I+II | 127 | ref |  |  |
| III+IV | 173 | 3.35 | <0.0001 | **** |
|  |  | (2.27-4.93) |  |  |
| **PTG_score** |  |  |  |  |
| low | 150 | ref |  |  |
| high | 150 | 3.27 | <0.0001 | **** |
|  |  | (2.29-4.69) |  |  |

* *P*<0.05, ** *P*<0.01, *** *P*<0.001, **** *P*<0.0001
